# Anterolateral Partial Sternotomy for Treatment of Graft Infection with Fungal Vegetation on the Frozen Elephant Trunk: A Case Report

**DOI:** 10.3400/avd.cr.21-00072

**Published:** 2021-12-25

**Authors:** Takanori Tsujimoto, Atsushi Omura, Takeshi Inoue, Syunya Chomei, Mari Hamaguchi, Taishi Inoue, Hidekazu Nakai, Katsuhiro Yamanaka, Kenji Okada

**Affiliations:** 1 Division of Cardiovascular Surgery, Department of Surgery, Kobe University Graduate School of Medicine, Kobe, Hyogo, Japan

**Keywords:** anterolateral partial sternotomy, graft infection, frozen elephant trunk

## Abstract

A 49-year-old man, who had undergone total arch replacement (TAR) with frozen elephant trunk (FET) technique for type A acute aortic dissection, was subsequently transferred to our hospital for uncontrollable infection. Since multiple blood cultures were positive for *Candida parapsilosis* and transesophageal echocardiography revealed vegetation attached to the FET, he was diagnosed with a graft infection. In addition, on the 18-fluorodeoxyglucose positron emission tomography scans, high uptake lesions were found around the quadrifurcated graft as well as the FET. Therefore, an extensive TAR through anterolateral thoracotomy with partial sternotomy was performed to remove all infected prothesis. Consequently, the patient completely recovered.

## Introduction

Frozen elephant trunk (FET) technique is being increasingly used during the primary treatment of type A acute aortic dissection since it brings satisfactory results early on while reducing the need for additional repair of the residual aorta.^1,2)^ On the other hand, systemic infection when extended to FET is rare but can result in a devastating outcome. Herein, we demonstrate effective approaches in diagnosing FET infections and to radical treatment. The patient provided informed consent for the presentation and publication of his case. This study has been approved by the ethical committee of our institution (approval number: no. B190201).

## Case Report

A 47-year-old man had been first admitted to a local hospital with a complaint of chest pain and was diagnosed with Stanford type A aortic dissection. He had received emergent total arch replacement (TAR) using a 22-mm quadrifurcated woven Dacron graft with FET technique using J Graft open stent graft (Japan Lifeline Co., Ltd., Tokyo, Japan), a 23-mm installation that was 9-cm long. Although the patient had experienced several severe complications, including reexploration, cerebral infarction, and tracheostomy, he had eventually recovered and been discharged.

Two years after the first admission, he presented to the same hospital with fever and general fatigue that had persisted for 2 months and was then transferred to our hospital due to an uncontrollable infection and acute renal failure.

**Figure 1:**
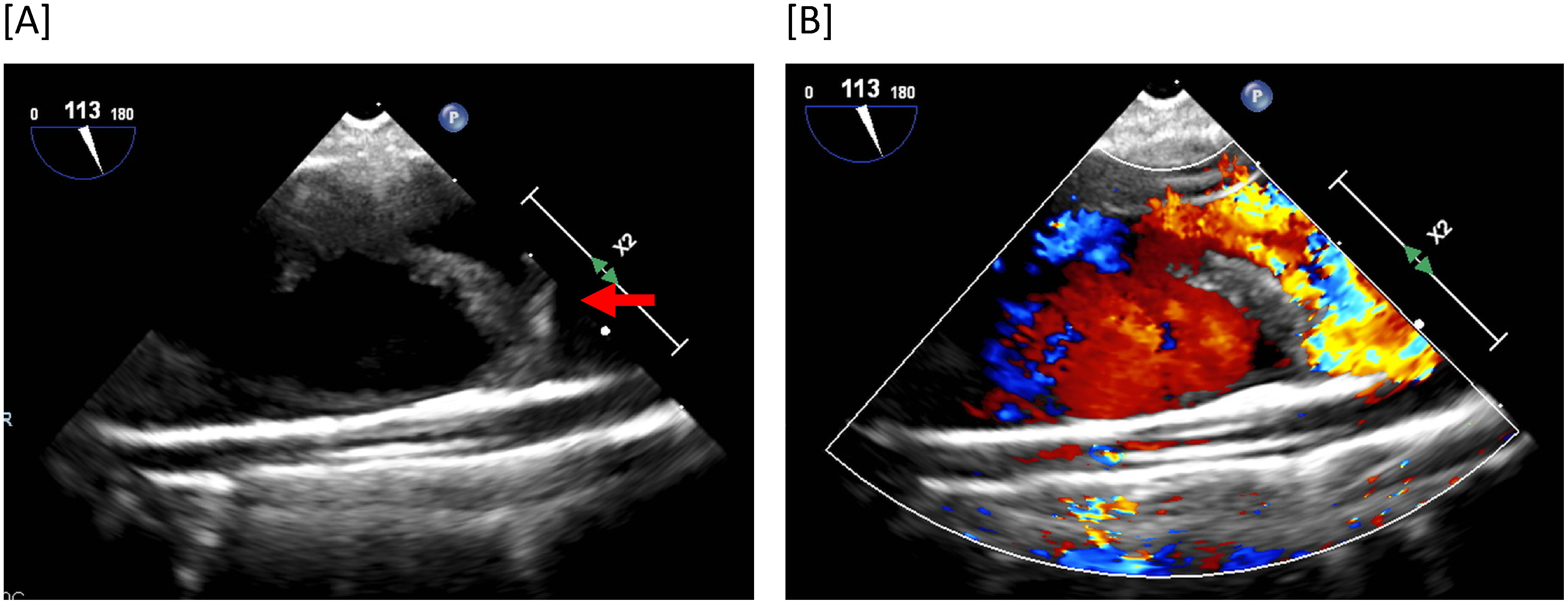
Fig. 1 (**A**) Preoperative TEE revealed mobile vegetation in FET (long axis view). (**B**) A mosaic flow pattern was observed in proximal side of the FET.

TEE: transesophageal echocardiography; FET: frozen elephant trunk

**Figure 2:**
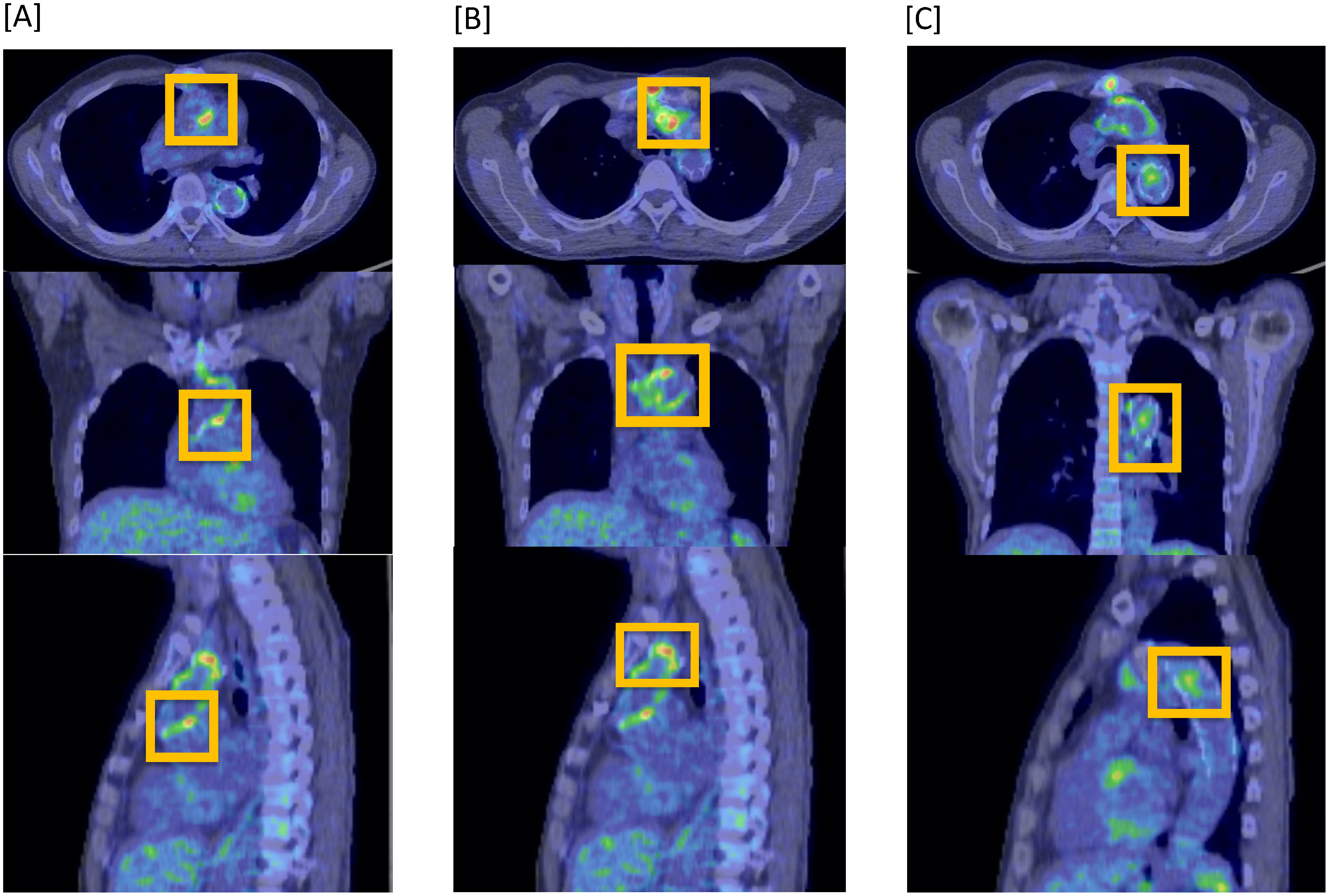
Fig. 2 Fusional FDG PET/CT images. From top to bottom, the axial view, coronal view, and sagittal view are listed. Orange rectangles: high uptake area on FDG PET/CT.

Since multiple blood cultures were positive for *Candida parapsilosis* despite intravenous administration of micafungin sodium and transesophageal echocardiography (TEE) revealed vegetation (**Figs. 1A** and **1B**) on the proximal side of the FET, the patient was diagnosed with a graft infection. Therefore, 18-fluorodeoxyglucose positron emission tomography/computed tomography (FDG PET/CT) was performed in order to identify appropriate extent of infected vascular prosthesis. On FDG PET/CT, high uptake lesions were found around the ascending aortic graft (**Fig. 2A**), the aortic arch graft (**Fig. 2B**), as well as the proximal side of the FET (**Fig. 2C**). The maximal standardized uptake values (SUV max) in these areas were 6.11, 6.48, and 4.98, respectively. Accordingly, the entire replacement of the prior prothesis consisted of the quadrifurcated graft, and FET was performed on day 4 of admission after routine examinations.

High uptake lesions around the ascending aortic graft (**A**), the aortic arch graft (**B**), the proximal side of the FET (**C**). FDG PET/CT: 18-fluorodeoxyglucose positron emission tomography/computed tomography; FET: frozen elephant trunk

**Figure 3:**
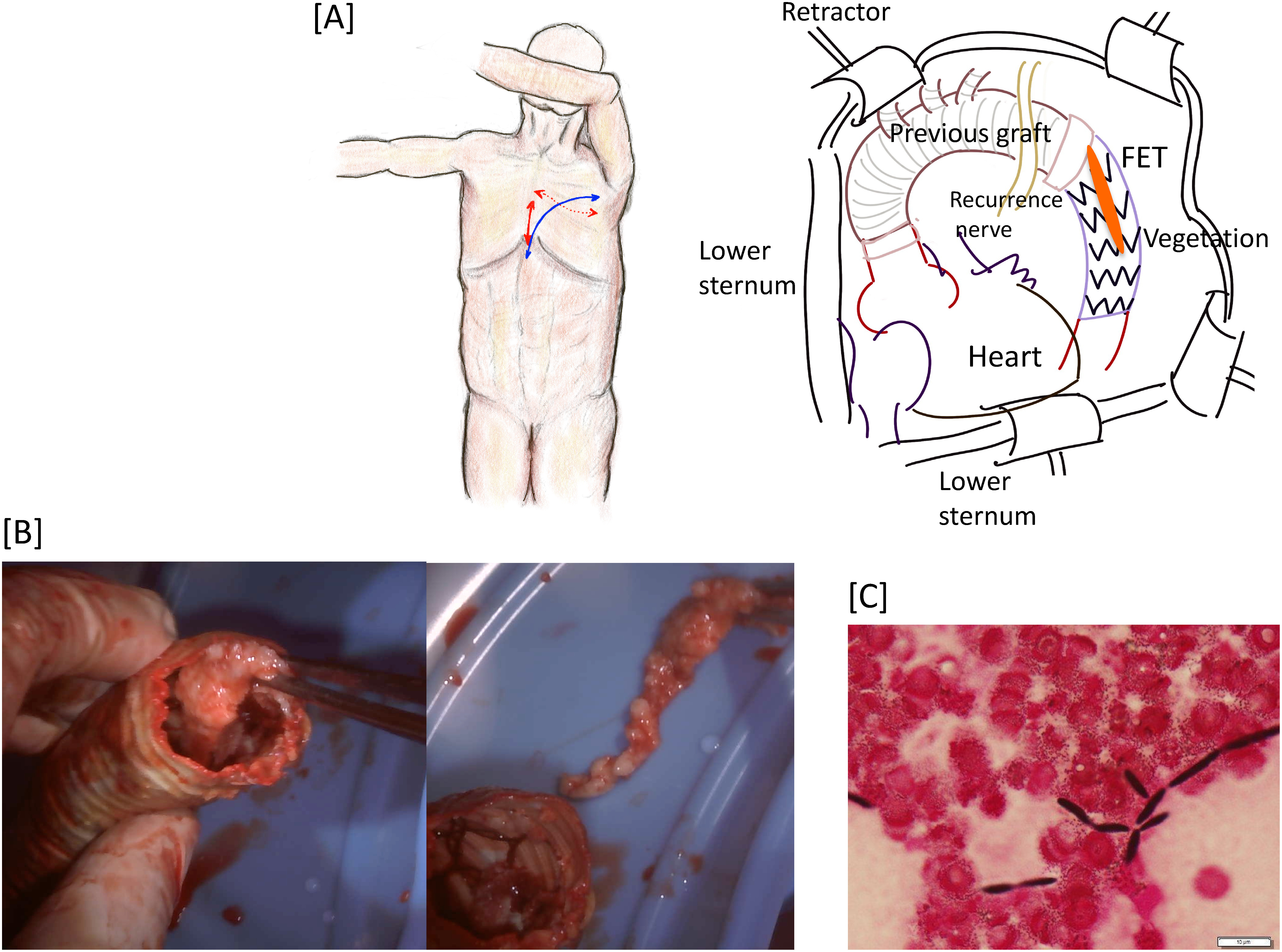
Fig. 3 (**A**) Schematic representation of ALPS approach and its surgical field. Red line: lower sternum and the third intercostal space. Blue line: skin incision which was extended from the anterior axillary line on the left to the xiphoid. (**B**) Removed FET with vegetation. The large vegetation was attached to the proximal side of FET and collapsed when grasped with forceps, due to its fragility. (**C**) A large number of fungal specimens with positive Grocott staining.

ALPS: anterolateral thoracotomy with partial sternotomy; FET: frozen elephant trunk

Firstly, the entire prosthesis was exposed through anterolateral thoracotomy with partial sternotomy (ALPS) through the third intercostal space (**Fig. 3A**), and dense adhesion surrounded the entire graft. Secondly, the inside of the prosthesis was also exposed, and the FET, to which long fragile vegetation had become attached, was removed (**Fig. 3B**). A large number of fungal specimens with positive Grocott staining were found in the vegetation (**Fig. 3C**).

Thirdly, the previous graft was completely replaced with a rifampicin-impregnated 22-mm woven Dacron graft (J Graft).

Lastly, the chest was routinely closed after omentum flap grafting over the ascending and arch parts of the new graft. The extracorporeal circulation time was 232 min, myocardial ischemia time was 92 min, selective cerebral perfusion time was 119 min, and operation time was 15 h and 14 min.

Following intravenous administration of antifungal drugs for 6 weeks, an oral antifungal drug (voriconazole, 150 mg/day) was prescribed for lifelong administration to prevent recurrent infection. Although temporary tracheostomy was necessary, the patient was moved to the general ward on day 26 of surgery and was eventually discharged 3 months after the surgery.

## Discussion

Doscher et al.^3)^ reported that intravascular graft infections are extremely rare; however, the disease is clinically important because it attacks patients regardless of age and results in a fatal outcome in cases of inaccurate diagnosis or treatment. Previously, we demonstrated a rare case of intravascular graft infection. In this report, vegetation was attached to an elephant trunk (ET) detected by TEE,^4)^ which likely occurred from vena contracta at the distal end of the ET caused by a discrepancy of diameter between the descending aorta and the ET.^5)^ On the other hand, since vegetation with a mosaic flow pattern was detected by TEE on the proximal side of FET in the current case (**Fig. 1**), a discrepancy at the distal anastomosis between an arch graft and non-stent part of FET was potentially related to the establishment of infection.

Mortality of intravascular graft infection was extremely high especially when its causative microorganism is fungus (41%, 5/12).^3)^ Survival depends on the entire excision of the infected graft as well as the appropriate administration of antifungal drugs.

Although conventional computed tomography (CT) scanning is gold standard in the diagnosis of aortic graft infection,^6)^ FDG PET/CT has recently been used to identify both infections and their anatomic localization.^6,7)^ Typical features of graft infection seen in CT, such as perigraft air, fluid, and soft-tissue attenuation, were cloaked in this case. Contrastingly, FDG PET/CT revealed an accumulation of FDG in the proximal and arch part of prosthetic graft as well as FET (**Fig. 2**). Intraoperative culture tests for samples from all part of the graft and FET revealed the presence of infection by *Candida parapsilosis*, proving that the diagnosis was correct.

When it comes to surgical approach, ALPS allows safe establishment of extracorporeal circulation (ECC), including left ventricular venting, ensures antegrade selective cerebral perfusion (SCP), and provides the surgical field to view the whole thoracic aorta.^8,9)^ Although an extended left thoracotomy incision with sternal transection also provides a fine surgical field for extensive thoracic aortic repair,^10)^ ALPS is considered as a more appropriate approach for this case because it often provides a better view of the cranial side, bringing advantage to prevent cerebral infarction caused by the vegetation collapsing. On the whole, ALPS is considered as a superior surgical approach to facilitate safe performance of such a complicated aortic procedure.

## Conclusion

We believe this is a noteworthy report describing a patient with vegetation on the FET, who underwent radical removal of the entire vascular prosthesis and fully recovered without recurrent infection. ALPS approach is an effective way to carry out an extensive aortic replacement from the ascending aorta to descending alternative, realizing safe ECC and SCP establishment. In addition, TEE is useful for detecting vegetation on the FET, and FDG PET/CT can help identify the extent of infected aortic graft.
